# Application of machine learning model to predict osteoporosis based on abdominal computed tomography images of the psoas muscle: a retrospective study

**DOI:** 10.1186/s12877-022-03502-9

**Published:** 2022-10-13

**Authors:** Cheng-bin Huang, Jia-sen Hu, Kai Tan, Wei Zhang, Tian-hao Xu, Lei Yang

**Affiliations:** 1 Department of Orthopaedic Surgery, The Second Affiliated Hospital and Yuying Childrens Hospital of Wenzhou Medical University, Wenzhou, 325000 China; 2 Key Laboratory of Orthopaedics of Zhejiang Province, Wenzhou, 325000 China

**Keywords:** Osteoporosis, Psoas, Machine learning, Computed tomography, Radiomics, Middle-aged and aged people

## Abstract

**Background:**

With rapid economic development, the world's average life expectancy is increasing, leading to the increasing prevalence of osteoporosis worldwide. However, due to the complexity and high cost of dual-energy x-ray absorptiometry (DXA) examination, DXA has not been widely used to diagnose osteoporosis. In addition, studies have shown that the psoas index measured at the third lumbar spine (L3) level is closely related to bone mineral density (BMD) and has an excellent predictive effect on osteoporosis. Therefore, this study developed a variety of machine learning (ML) models based on psoas muscle tissue at the L3 level of unenhanced abdominal computed tomography (CT) to predict osteoporosis.

**Methods:**

Medical professionals collected the CT images and the clinical characteristics data of patients over 40 years old who underwent DXA and abdominal CT examination in the Second Affiliated Hospital of Wenzhou Medical University database from January 2017 to January 2021. Using 3D Slicer software based on horizontal CT images of the L3, the specialist delineated three layers of the region of interest (ROI) along the bilateral psoas muscle edges. The PyRadiomics package in Python was used to extract the features of ROI. Then Mann–Whitney U test and the least absolute shrinkage and selection operator (LASSO) algorithm were used to reduce the dimension of the extracted features. Finally, six machine learning models, Gaussian naïve Bayes (GNB), random forest (RF), logistic regression (LR), support vector machines (SVM), Gradient boosting machine (GBM), and Extreme gradient boosting (XGBoost), were applied to train and validate these features to predict osteoporosis.

**Results:**

A total of 172 participants met the inclusion and exclusion criteria for the study. 82 participants were enrolled in the osteoporosis group, and 90 were in the non-osteoporosis group. Moreover, the two groups had no significant differences in age, BMI, sex, smoking, drinking, hypertension, and diabetes. Besides, 826 radiomic features were obtained from unenhanced abdominal CT images of osteoporotic and non-osteoporotic patients. Five hundred fifty radiomic features were screened out of 826 by the Mann–Whitney U test. Finally, 16 significant radiomic features were obtained by the LASSO algorithm. These 16 radiomic features were incorporated into six traditional machine learning models (GBM, GNB, LR, RF, SVM, and XGB). All six machine learning models could predict osteoporosis well in the validation set, with the area under the receiver operating characteristic (AUROC) values greater than or equal to 0.8. GBM is more effective in predicting osteoporosis, whose AUROC was 0.86, sensitivity 0.70, specificity 0.92, and accuracy 0.81 in validation sets.

**Conclusion:**

We developed six machine learning models to predict osteoporosis based on psoas muscle images of abdominal CT, and the GBM model had the best predictive performance. GBM model can better help clinicians to diagnose osteoporosis and provide timely anti-osteoporosis treatment for patients. In the future, the research team will strive to include participants from multiple institutions to conduct external validation of the ML model of this study.

## Introduction

Osteoporosis is a systemic bone disease caused by decreased bone density and quality, the destruction of bone microstructure, and increased bone fragility [1, 2]. With the rapid economic development, the world's average life expectancy is increasing, which leads to the increasing prevalence of osteoporosis in the world [3]. Patients with osteoporotic fractures have poorer functional recovery and even increased mortality compared with non-osteoporotic fractures [4, 5]. The gold standard for clinical diagnosis of osteoporosis is bone mineral density (BMD) and t score measured by dual-energy x-ray absorptiometry (DXA) [6]. However, DXA is not a routine test because of its high cost. Therefore, there is an urgent need for a simple and efficient method to screen osteoporosis patients in advance.

Sarcopenia is a syndrome of progressive muscle mass, strength, and muscle function loss with age [7]. Skeletal muscle mass in adults older than 40 declines by about 1% annually [8]. More and more studies have shown that sarcopenia is associated with osteoporosis, and proper muscle exercise can effectively prevent osteoporosis [9–11]. In addition, studies have shown that the psoas index measured at the third lumbar spine (L3) level is closely related to BMD and has an excellent predictive effect on osteoporosis [12].

Machine learning (ML) models are widely used in the medical field due to their excellent performance in predicting classification problems [13, 14]. Some studies have shown that applying ML models based on X-ray or vertebral computed tomography (CT) images can effectively predict osteoporosis [15, 16]. However, there are currently no studies to apply machine learning models based on muscle tissue to predict osteoporosis. Therefore, this study developed various machine learning models based on psoas muscle tissue at the third lumbar spine (L3) level of unenhanced abdominal CT to predict osteoporosis, thus providing some help for clinical screening of patients with osteoporosis.

## Materials and methods

### Study population

**Fig. 1:**
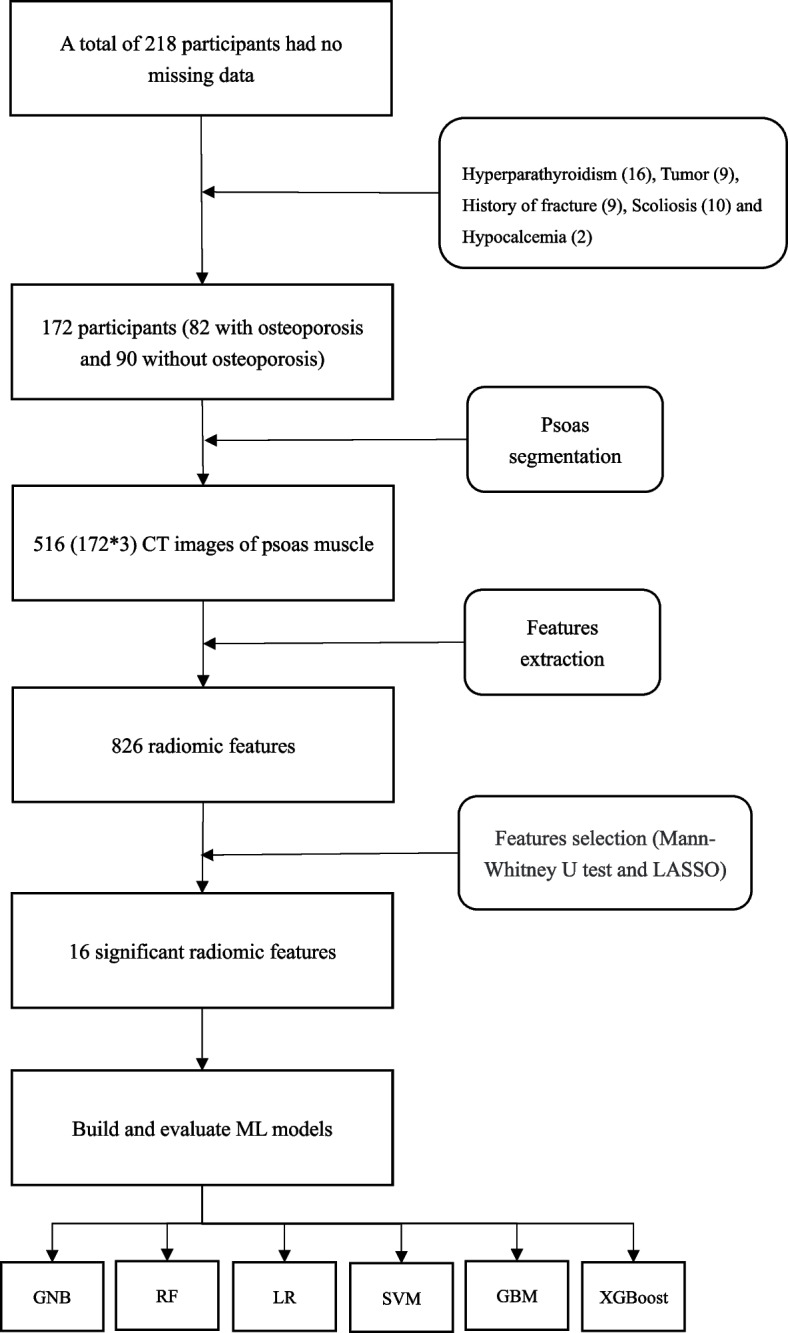
Flow chart showing analyses and model making process for the study. Abbreviations: ML, machine learning; CT, computed tomography; LASSO, least absolute shrinkage and selection operator; LR, Logistic regression; GBM, Gradient boosting machine; RF, Random forest; GNB, Gaussian naïve Bayes; XGBoost, Extreme gradient boosting; SVM, Support vector machines

We retrospectively collected patients over 40 years old from the Department of Endocrinology, the Second Affiliated Hospital of Wenzhou Medical University, from January 2017 to January 2021. The inclusion criteria were:1) The interval between unenhanced abdominal CT and DXA (lumbar spine and femoral neck) was less than three months, and 2) Age ≥ 40 years. The exclusion criteria were: 1) No unenhanced abdominal CT and DXA and 2) Previous history of hyperparathyroidism, tumor, hypocalcemia and fracture affecting the bones and muscles. According to European clinical guidelines [6], patients with a lumbar (L1-4) or femoral neck T-score of less than -2.5 are diagnosed with osteoporosis and patients with both lumbar and femoral neck T scores above -2.5 are non-osteoporosis. Figure 1 shows the flow chart of this research method.

### Psoas segmentation

**Fig. 2:**
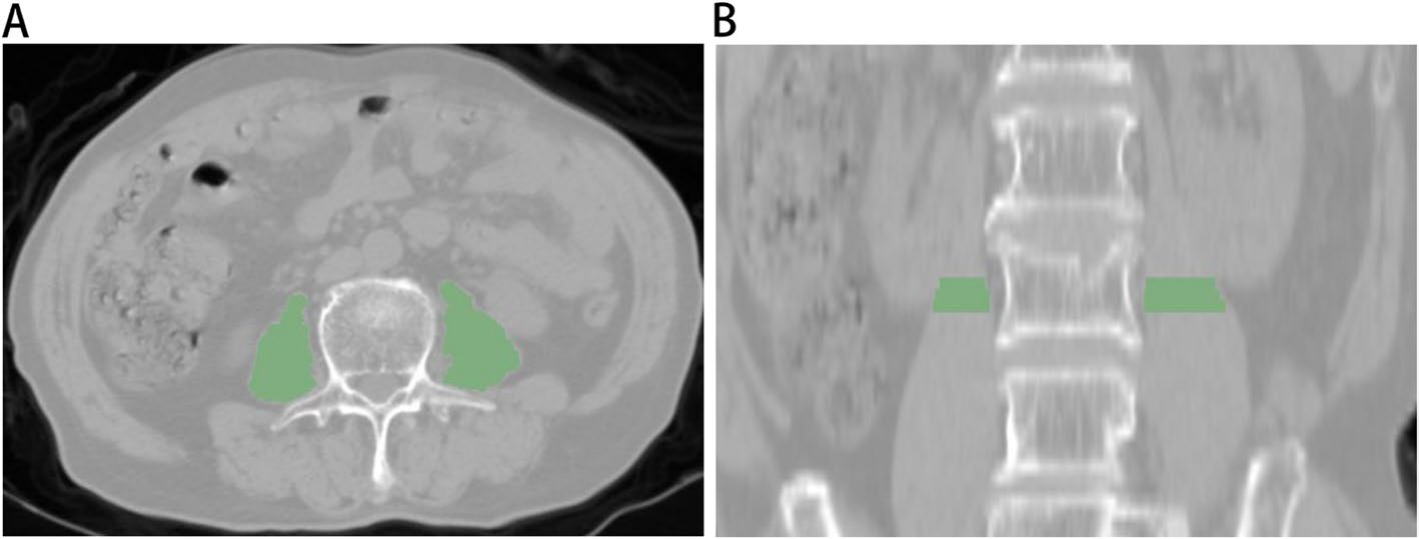
The psoas muscle at the third lumbar level was segmented in **A**) transverse plane and **B**) coronal plane

Unenhanced abdominal CT data were obtained by the picture archiving and communication system (Philips) operated at 120 kV and 250 mA with a slice thickness of 5 mm. In addition, the CT data were obtained after the DXA examination within three months. Using 3D Slicer (version 5.0.3) software based on horizontal CT images of the third lumbar spine, the specialist delineated three layers of the region of interest (ROI) along the bilateral psoas muscle edges. (Fig. 2). The computer automatically generates the volume of interest (VOI) of lesions. Another specialist checked the contour results. These two experts have at least 5 years of experience in clinical work and are skilled in using 3D Slicer software, which can well outline the psoas muscle of the participants. Moreover, neither expert knew how the participants were grouped.

### Features extraction and selection

Feature extraction is performed using the PyRadiomics [17] package in Python. A total of 826 radiomics features were extracted for each patient. Features were divided into six groups: (1) First-order statistics of psoas (*n* = 18), (2) shape (*n* = 14), (3) texture (*n* = 24, derived from GLCM), (4) texture (*n* = 16, derived from GLRLM), (5) wavelet-based features (*n* = 464), and (6) Laplacian of Gaussian-filtered image features (*n* = 290). Each feature was named by concatenating the image type from which the feature was extracted, feature group, and feature name by underline. For example, 'original_glcm_Idmn was a feature extracted from the original image, GLCM group, and the name was Idmn.

### Statistical analysis

Clinical baseline characteristics data distribution was tested using the Shapiro–Wilk test. As appropriate, patient characteristics were described using mean ± standard deviation, frequency, and percentage. Normally distributed variables were analyzed using Student’s t-test. Categorical variables were expressed as percentages and analyzed using the Pearson Chi-squared test. All statistics were calculated using SPSS software (version 26.0; SPSS Inc., Chicago, IL, USA).

Firstly, the extracted radiomic features were screened by the Mann–Whitney U test, and extracted radiomic features with *P* < 0.05 were screened. Second, the radiomic features with *P* < 0.05 were standardized using the StandardScaler function. Then, the alpha parameter with the minimum mean square error is obtained through 1000 iterations after fivefold cross-validation based on standardized features. Based on the optimal alpha parameter, the least absolute shrinkage and selection operator (LASSO) feature selection algorithm is used to select the relevant features and calculate the coefficients of each feature. Moreover, the radiation characteristics of non-zero coefficients are obtained. The LASSO algorithm can reduce features' dimensions and screen out the most meaningful feature effects. Finally, the meaningful radiomics features screened by the Mann–Whitney U test and LASSO algorithm were put into the machine learning model for prediction. We randomly split our dataset into two groups: the training sets (60%) for ML model development and the validation sets (40%) for performance evaluation. Besides, we applied six supervised machine learning algorithms: Gaussian naïve Bayes (GNB), random forest (RF), logistic regression (LR), support vector machines (SVM), Gradient boosting machine (GBM), and Extreme gradient boosting (XGBoost). Furthermore, we evaluated the predictive ability of each ML classifier in validation sets where the area under the receiver operating characteristic (AUROC) value and the corresponding sensitivity, specificity, and overall accuracy of ML algorithms were all calculated. These classifiers were imported from a Python (version 3.7.6) machine learning library called scikit-learn. In addition, this study's most important outcome measure was whether the participants had osteoporosis, which the ML model predicted.

## Results

### Participants

A total of 172 participants met the inclusion and exclusion criteria for the study. Based on the T-scores of the femoral neck and lumbar spine examined by DXA, 82 participants were enrolled in the osteoporosis group, and 90 were enrolled in the non-osteoporosis group. Table 1 presents the clinical baseline characteristics of the two groups of participants. There were no significant differences between the two groups in age, BMI, sex, smoking, drinking, hypertension, and diabetes.

**Table 1:** Comparison of clinical characteristics between two groups

	**Non-osteoporosis (90)**	**osteoporosis(82)**	***P* value**
Age (years)	62 ± 9	63 ± 13	0.448
BMI (kg/m^2^)	24.07 ± 3.02	23.31 ± 3.63	0.136
Gender			0.432
Female, n(%)	59(65.6)	49(59.8)	
Male, n(%)	31(34.4)	33(40.2)	
Hypertension, n(%)	57(63.3)	42(51.2)	0.108
Diabetes, n(%)	65(72.2)	64(78.0)	0.378
Current drinking, n(%)	10(11.1)	11(13.4)	0.645
Current smoking, n(%)	12(13.3)	12(14.6)	0.806

*Abbreviations*: *BMI* body mass index

### Feature selection of radiomics

**Fig. 3:**
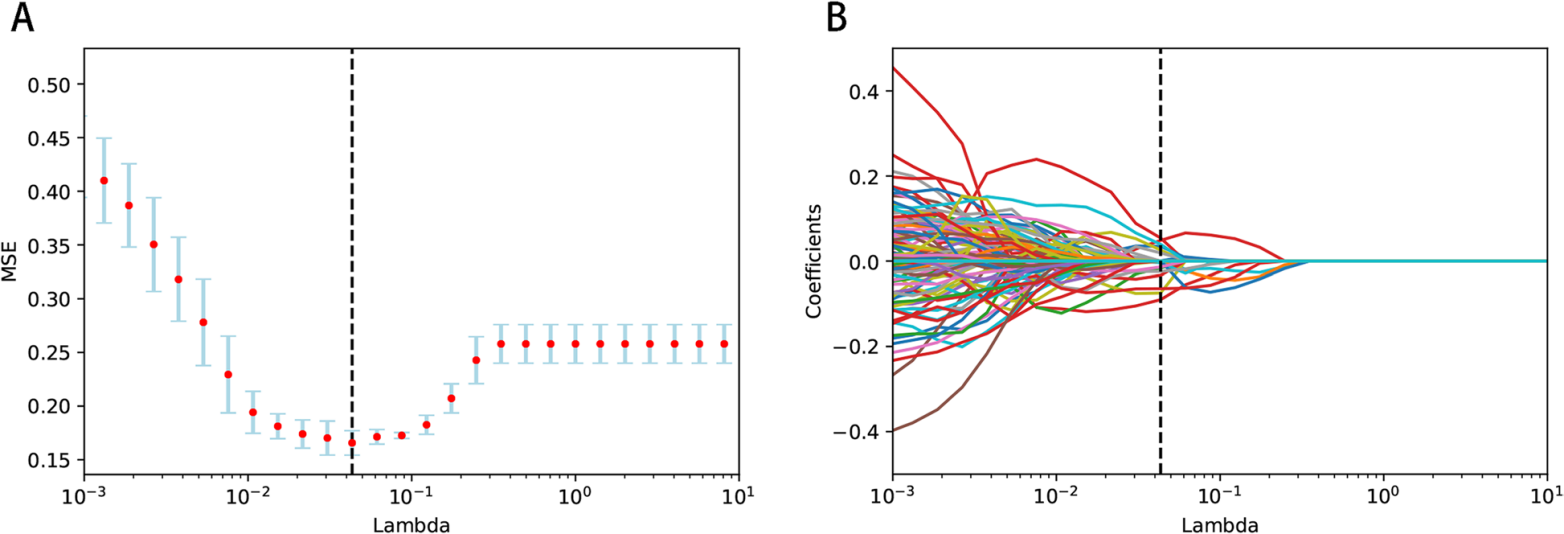
The least absolute shrinkage and selection operator algorithm was applied to select features

Eight hundred twenty-six radiomic features were obtained from unenhanced abdominal CT images of osteoporotic and non-osteoporotic patients. Five hundred fifty radiomic features were screened out of 826 by the Mann–Whitney U test. The optimal alpha parameter of psoas muscle image features is about 0.043 (Fig. 3). Based on the optimal alpha parameter, the LASSO algorithm was used to reduce the dimension of the above high-dimensional features and screen out the best features. Sixteen radiomic features were obtained, including 'log-sigma-1–0-mm-3D_firstorder_Uniformity', 'log-sigma-1–0-mm-3D_glcm_JointEnergy', 'log-sigma-1–0-mm-3D_glcm_MaximumProbability', 'log-sigma-2–0-mm-3D_glcm_Imc2', 'log-sigma-3–0-mm-3D_glcm_Imc1', 'log-sigma-5–0-mm-3D_firstorder_90Percentile', 'log-sigma-5–0-mm-3D_firstorder_MeanAbsoluteDeviation', 'log-sigma-5–0-mm-3D_firstorder_Skewness', 'log-sigma-5–0-mm-3D_glcm_ClusterShade', 'original_firstorder_Range', 'original_glcm_Idmn', 'original_glcm_MCC', 'wavelet-HLH_glrlm_ShortRunLowGrayLevelEmphasis', 'wavelet-HLL_glcm_Autocorrelation', 'wavelet-HLL_glrlm_ShortRunLowGrayLevelEmphasis' and 'wavelet-LLH_firstorder_Median.

### Diagnostic performance of radiomics models

**Fig. 4:**
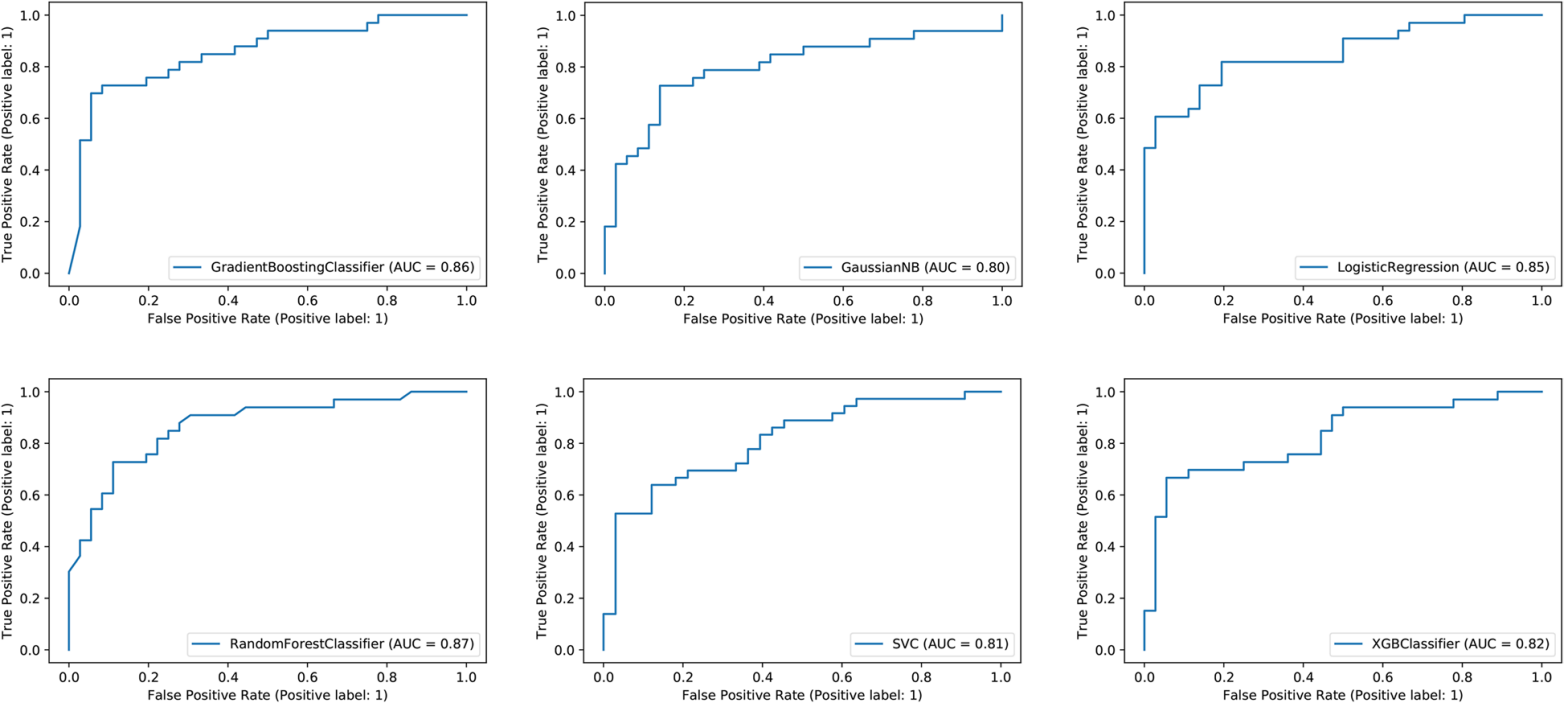
ROC curve analysis of machine learning algorithms for prediction of osteoporosis patients in the validation set. Abbreviations: LR, Logistic regression; GBM, Gradient boosting machine; RF, Random forest; GNB, Gaussian naïve Bayes; XGBoost, Extreme gradient boosting; SVM, support vector machines; ROC, receiver operating characteristic; AUC, area under the curve

These 16 radiomic features were incorporated into six traditional machine learning models (GBM, GNB, LR, RF, SVM, and XGB). All six machine learning models could predict osteoporosis well in the validation set, with AUROC values greater than or equal to 0.8 (Fig. 4). In addition, Table 2 presents the relevant evaluation indexes (AUROC, sensitivity, specificity, and accuracy) of the effectiveness of the six machine learning models in predicting osteoporosis. GBM is more effective in predicting osteoporosis, whose AUROC was 0.86, sensitivity 0.70, specificity 0.92, and accuracy 0.81 in validation sets. In addition, Table 3 presents the specific parameters of the six ML models in this study. In this study, we only adjusted some parameters in the ML model, and most parameters were still the default parameters. For example, in the SVM model, we use GridSearch [18] to obtain the best parameters C (C = 2.33) and gamma (2.15e-04).

**Table 2:** Predictive performance comparison of the five types of machine learning algorithms in the validation sets

Model	AUROC	Sensitivity	Specificity	Accuracy
LR	0.85	0.73	0.86	0.80
XGBoost	0.82	0.70	0.75	0.72
GNB	0.80	0.73	0.86	0.80
GBM	0.86	0.70	0.92	0.81
RF	0.87	0.73	0.86	0.80
SVM	0.81	0.86	0.55	0.71

*Abbreviations*: *LR* Logistic regression, *GBM* Gradient boosting machine, *RF* Random forest, *GNB* Gaussian naïve Bayes, *XGBoost* Extreme gradient boosting, *SVM* Support vector machines, AUROC area under the receiver operating characteristic

**Table 3:** parameters of all machine learning models in this study

Model	parameters
LR	penalty = 'l2', dual = False, tol = 0.0001, C = 1.0, fit_intercept = True, intercept_scaling = 1, class_weight = None, random_state = None, solver = 'lbfgs', max_iter = 100, multi_class = 'auto', verbose = 0, warm_start = False, n_jobs = None, l1_ratio = None
XGBoost	n_estimators = 500, learning_rate = 0.5, objective = 'binary:logistic', use_label_encoder = True
GNB	priors = None, var_smoothing = 1e-09
GBM	loss = 'deviance', learning_rate = 0.5, n_estimators = 500, subsample = 1.0, criterion = 'friedman_mse', min_samples_split = 2, min_samples_leaf = 1, min_weight_fraction_leaf = 0.0, max_depth = 3, min_impurity_decrease = 0.0, min_impurity_split = None, init = None, random_state = None, max_features = None, verbose = 0, max_leaf_nodes = None, warm_start = False, validation_fraction = 0.1, n_iter_no_change = None, tol = 0.0001, ccp_alpha = 0.0
RF	n_estimators = 100, criterion = 'gini', max_depth = None, min_samples_split = 2, min_samples_leaf = 1, min_weight_fraction_leaf = 0.0, max_features = 'auto', max_leaf_nodes = None, min_impurity_decrease = 0.0, min_impurity_split = None, bootstrap = True, oob_score = False, n_jobs = None, random_state = None, verbose = 0, warm_start = False, class_weight = None, ccp_alpha = 0.0, max_samples = None
SVM	C = 2.33, kernel = 'rbf', degree = 3, gamma = 2.15e-04, coef0 = 0.0, shrinking = True, probability = False, tol = 0.001, cache_size = 200, class_weight = None, verbose = False, max_iter = -1, decision_function_shape = 'ovr', break_ties = False, random_state = None

*Abbreviations*: *LR* Logistic regression, *GBM* Gradient boosting machine, *RF* Random forest, *GNB* Gaussian naïve Bayes, *XGBoost* Extreme gradient boosting, *SVM* Support vector machines

## Discussion

With the increasing prevalence of osteoporosis worldwide, a single discipline is not an excellent way to prevent osteoporosis and treat its complications. More and more studies have shown that multidisciplinary management of osteoporosis patients, including nursing, endocrinology and geriatric medicine, can significantly reduce the burden on the social economy and health care. For elderly patients with hip fractures, timely management of nursing and other disciplines can shorten the length of hospital stay, reduce acute mortality and so on, thus significantly reducing society's medical burden society [19–21]. However, the most critical step in managing osteoporosis is the timely detection and diagnosis of osteoporosis patients. The gold criteria for the diagnosis of osteoporosis were BMD and T scores measured by DXA [6]. However, due to the complexity and high cost of DXA examination, DXA has not been widely used to diagnose osteoporosis. Therefore, more and more studies are trying to find a method to predict osteoporosis effectively and osteoporotic fractures. The level of bone turnover markers can reflect bone metabolism in the body. A high level of bone turnover markers can predict osteoporosis and osteoporotic fractures to a certain extent [22, 23]. Fracture risk assessment tool can effectively predict the probability of osteoporotic fractures in the next ten years [24]. In addition, the psoas muscle index can also predict osteoporosis to a certain extent [12]. In this study, we used multiple machine learning models to evaluate unenhanced abdominal psoas CT images and found that they were better than the psoas index in predicting osteoporosis. In addition, there was no significant difference in age, BMI and other clinical baseline characteristics between the osteoporosis group and the non-osteoporosis group in this study. Therefore, the results of this study excluded a series of confounding factors such as age and BMI, which made the ML model for predicting osteoporosis based on psoas CT images in this study reliable to a certain extent.

In recent years, with the development of computer technology, the ability of medical image processing is constantly improved. Texture analysis technology can extract quantitative data from medical images such as X-ray and CT images. The texture is an inherent property of surfaces in nature. Texture analysis refers to using image processing technology to analyze the intensity and distribution pattern of voxels or pixels in digital images and extract texture feature parameters to obtain quantitative features [25]. Therefore, texture analysis technology can detect data that the human eye cannot. The core of texture analysis is feature extraction, which quantitatively describes ROI attributes. In this study, we extracted a total of 826 texture features. Most of these 826 features are not statistically significant or have little weight in machine learning. In addition, the number of texture features was much greater than the number of patients. Therefore, to reduce the risk of overfitting, this study uses the Mann–Whitney U test and LASSO algorithm to reduce the dimension of feature data [26, 27]. Finally, 16 radiomics features were selected for subsequent machine learning model training and learning.

Machine learning is an essential branch of artificial intelligence. Machine learning models have been widely used in various fields, especially medicine, because of their powerful predictive ability for classification problems. Yupeng Zhang et al. [28] classified the causes of cerebral hematoma using ML models based on head CT images. Mutasa S et al. [29] used a deep learning model to classify femoral neck fracture types based on X-rays. Zhu J et al. [30] used a ML model to predict the presence or absence of lymph node metastasis in papillary thyroid carcinoma based on perioperative clinical baseline data. These ML models have achieved good results in classification, and to a certain extent, they can assist clinicians in providing better treatment for patients. Therefore, similar to the above studies, this study used ML models to predict the classification problem of osteoporosis in middle-aged and older adults. For training and learning, the last 16 radiomics features were put into six traditional ML models (LR, XGBoost, GNB, GBM, RF, and SVM). The best model for predicting osteoporosis was selected from these six ML models for further research in the future.

Many studies have explored the relationship between skeletal muscle and osteoporosis. The relationship between skeletal muscle and bone is not only mechanical. As endocrine organs, skeletal muscle and bone produce various cytokines, such as interleukin and irisin, which affect the growth and differentiation of osteogenic and osteoclast cells, thus affecting the function of bone and muscle [31]. As a classical signaling pathway, the Receptor activator of the Nf-kb ligand (RANKL) is closely related to the pathophysiological mechanism of osteoporosis. Bonnet N. et al. [32] demonstrated that RANKL is closely associated with skeletal muscle function and that inhibition of RANKL activation can significantly improve muscle strength in patients with osteoporosis. In this study, the 6 ML model showed promising efficacy in predicting osteoporosis, with an AUROC of 0.80 or greater. These ML models have good predictive efficacy, indicating that abdominal CT examination of psoas muscle can predict osteoporosis. GBM model has the best predictive performance among the 6 ML models whose AUROC was 0.86, sensitivity 0.70, specificity 0.92, and accuracy 0.81 in validation sets. The GBM model has been proven robust in predicting performance in many studies in the medical field. Ji GW et al. [33] successfully developed a GBM model to predict the prognosis of patients with intrahepatic cholangiocarcinoma after surgery. Similarly, Seidler M et al. [34] applied the GBM model to distinguish normal lymph nodes from abnormal lymph nodes. In addition, the gradient enhancement algorithm based on CT images can accurately diagnose sarcopenia [35]. The results of these studies strongly support our findings that GBM is an efficient model for predicting osteoporosis based on abdominal CT images of the psoas muscle. Therefore, clinicians can use the GBM model based on abdominal CT psoas image to screen out patients at high risk of osteoporosis to diagnose osteoporosis before the occurrence of osteoporotic fracture and provide timely anti-osteoporosis intervention.

Several studies have applied various types of ML models to predict osteoporosis. Pan Y et al. [16] have successfully developed a deep learning model for predicting osteoporosis based on low-dose chest CT images. Zhang T et al. [36] developed an SVM model for predicting osteoporosis based on bone turnover markers. Shim JG et al. [37] developed various ML models to predict osteoporosis in postmenopausal women based on clinical baseline characteristics such as age and BMI. Similar to the above, this study also aims to apply the ML model to predict osteoporosis. However, the data used in this study are quite different from those studies. Numerous studies have demonstrated that muscle mass is closely related to osteoporosis, especially psoas muscle mass at the L3 level. Therefore, this study is the first to apply multiple ML models to predict osteoporosis based on psoas CT images, and each ML model has achieved good predictive performance. The results of this study further support the close relationship between muscle and osteoporosis and provide a new, efficient and simple method to screen for osteoporosis.

However, there are some limitations to this study. First, although the machine learning model in this study achieved good performance, this study was a single-center retrospective study. Secondly, although LASSO and other methods were used in this study to avoid overfitting, the study's sample size was relatively small, and there was still the possibility of overfitting. Thirdly, the different models of CT scanning equipment used by different institutions may lead to uneven image quality, affecting the results of this study. Therefore, a multicenter prospective study with a large sample size is needed to support the results of this study. In addition, some confounding factors, such as age, were excluded from this study. However, some confounding factors were still not included in this study, such as rehabilitation and pain treatment history. Finally, the study was conducted on subjects over 40 years of age, so the results of this study do not apply to patients with idiopathic osteoporosis. Due to the above limitations, the ML model in this study may not apply to all patients. A future study is needed to investigate this in depth.

## Conclusion

In this study, we developed six machine learning models to predict osteoporosis based on psoas muscle images of abdominal CT, and the GBM model had the best predictive performance. GBM model can better help clinicians to diagnose osteoporosis and provide timely anti-osteoporosis treatment for patients. In the future, the research team will strive to include participants from multiple institutions to conduct external validation of the ML model of this study.

## Data availability declaration

The datasets analyzed in the study are available from the corresponding author on reasonable request.

## Abbreviations

ML: Machine learning
DXA: Dual-energy x-ray absorptiometry
BMD: Bone mineral density
L3: Third lumbar spine
CT: Computed tomography
ROI: Region of interest
VOI: Volume of interest
LASSO: Least absolute shrinkage and selection operator
LR: Logistic regression
GBM: Gradient boosting machine
RF: Random forest
GNB: Gaussian naïve Bayes
XGBoost: Extreme gradient boosting
SVM: Support vector machines
AUROC: Area under the receiver operating characteristic
RANKL: Receptor activator of Nf-kb ligand
